# Urodynamic Evaluation after High-Intensity Focused Ultrasound for Patients with Prostate Cancer

**DOI:** 10.1155/2014/462153

**Published:** 2014-05-15

**Authors:** Luigi Mearini, Elisabetta Nunzi, Silvia Giovannozzi, Luca Lepri, Carolina Lolli, Antonella Giannantoni

**Affiliations:** Department of Urology and Andrology, University of Perugia, Sant'Andrea delle Fratte, 06100 Perugia, Italy

## Abstract

This prospective study assesses the impact of high-intensity focused ultrasound (HIFU) on lower urinary tract by comparing pre- and postoperative symptoms and urodynamic changes. Thirty consecutive patients with clinically organ-confined prostate cancer underwent urodynamic study before HIFU and then at 3–6 months after surgery. Continence status and symptoms were analyzed by means of International Prostate Symptoms Score IPSS and International Index Erectile Function IIEF5. As a result, there were a significant improvement in bladder outlet, maximum flow at uroflowmetry, and reduction in postvoid residual PVR at 6-month follow-up and a concomitant significant reduction of detrusor pressure at opening and at maximum flow. De novo overactive bladder and impaired bladder compliance were detected in 10% of patients at 3 months, with progressive improvement at longer follow-up. Baseline prostate volume and length of the procedure were predictors of 6-month IPSS score and continence status. In conclusion, following HIFU detrusor overactivity, decreased bladder compliance and urge incontinence represent de novo dysfunction due to prostate and bladder neck injury during surgery. However, urodynamic study shows a progressive improvement in all storage and voiding patterns at 6-month follow-up. Patients with high prostate volume and long procedure length suffered from irritative symptoms even at long term.

## 1. Introduction

The European Association of Urology and the American Urological Association recommend radical prostatectomy (RP) or external-beam radiotherapy (EBRT) as the standard treatment options for patients with localized prostate cancer [1].

Despite the recent developments in surgical techniques with the introduction of robot-assisted RP [2] and new radiotherapy devices [3], urinary incontinence and erectile dysfunction continue to be the most devastating complications following radical treatment. Active surveillance spares continence and potency in cases of low-risk prostate cancer; however, some patients have radical treatments as a result of disease progression or psychological distress [4]. Thus, a minimally invasive (albeit investigational) therapy such as high-intensity focused ultrasound (HIFU) offers the possibility of a cure with reduced side effects for selected patients.

In HIFU, ultrasound beams emitted from a high-powered transducer target a precise tissue volume sparing the surrounding tissue. Because HIFU is a minimally invasive procedure and comparable to a curative therapy, we expected to find a high curative efficacy and low incidence of incontinence and impotence, as well as small effects on the lower urinary tract (LUT).

Perioperative and long-term side effects following HIFU have been extensively described; the most common include urinary retention, urinary tract infections, incontinence, and erectile dysfunction.

In particular, the rates of urinary retention ranged from <1% to 20% [5]. Acute urinary retention is an expected effect of thermal injury, edema, and swelling of the prostate [6], with the prostate volume increasing up to 30% from baseline. A transurethral resection of prostate (TURP) prior to HIFU and the insertion of a catheter or suprapubic tube are the simplest ways to prevent or to treat acute urinary retention [7].

Sloughing, the elimination of necrotic tissue, is another LUT problem. During sloughing, patients complain of dysuria with urgency as well as irritative or obstructive symptoms, or both. A preoperative TURP or symptomatic treatment using drugs is usually sufficient.

Another frequent complication is bladder outlet obstruction (BOO) as a result of bladder neck, urethral, or both types of stenosis; BOO occurs in 3.6% to 24.5% of all cases [8], and this complication is usually managed by dilation. Only a few cases require surgical procedures such as transurethral incision or TURP.

To date, no study has analyzed LUT function using urodynamics before and after primary HIFU to treat localized prostate cancer.

Moreover, the common occurrence of side effects in the LUT is correlated with different ablative technologies and different prostate gland approaches.

The current prospective study investigated the clinical and urodynamic patterns of patients treated with HIFU.

## 2. Materials and Methods

### 2.1. Demographics

Thirty consecutive patients with clinically localized prostate cancer (cT1c-cT2c) undergoing HIFU were prospectively enrolled in this study, which was conducted in accordance with the Declaration of Helsinki (1964) and approved by internal review board.

The inclusion criteria for HIFU were a histological diagnosis of prostate cancer, a PSA < 15 ng/mL, and stage T1c-T2 N0M0 (N and M statuses were assessed using a CT scan and bone scintigraphy).

The exclusion criteria were a prostate volume greater than 50 cc (two treatments scheduled), the presence of a median lobe, intraprostatic calcification of more than 1 cm, or concomitant anal stricture.

No patients underwent TURP prior to HIFU or received neoadjuvant hormone therapy.

All patients were informed of the scientific nature of the investigation, and they provided written informed consent.

### 2.2. Surgical Technique

The HIFU technique adhered to the protocol described by Illing et al. [9, 10] in a previous paper [11].

This study used the Sonablate 500 (Focus Surgery, Indianapolis, IN, USA) HIFU device.

The HIFU probe has two focal lengths, with a focus length at 4.0 cm and 3.0 cm, which limited the treatable gland volume.

HIFU was performed without a preoperative TURP for all included cases. At the end of the procedure, a percutaneous cystostomy was inserted into patients to reduce the incidence of postoperative stenosis [12].

Follow-up assessments were scheduled at 1, 3, and 6 months and then every 6 months afterwards. These evaluations included an accurate objective examination using digital rectal examination (DRE), a transrectal ultrasound scan (TRUS) at 3 and 6 months, and urodynamics.

PSA levels were tested at 1, 3, and 6 months and then every 6 months afterwards. A prostate biopsy was performed at 6 months to obtain at least 8 samples depending on residual prostate volume.

### 2.3. Clinical and Urodynamic Evaluations

Clinical and urodynamic evaluations were performed 3–7 days before surgery (i.e., the baseline evaluation) and at 3–6 months after surgery.

A 1-month follow-up assessment was used to evaluate any LUT dysfunctions.

The clinical evaluation consisted of the patient's medical history, a physical examination, and the administration of the International Prostate Symptoms Score (IPSS) and the International Index of Erectile Function (IIEF-5).

A score of ≥7 on the IPSS indicates moderate-to-severe symptoms, whereas a score of ≤16 on the IIEF5 indicates moderate-to-severe erectile dysfunction.

To assess continence status, we recorded the number of daily pads (0-1 versus >1) and daily episodes of urgency as well as episodes of urgency and stress incontinence in a voiding diary.

Uroflowmetry with the detection of maximum flow (*Q*_max⁡_) and postvoid residual (PVR) volume was scheduled for all participants; the urodynamic evaluation was performed in patients without urinary tract infections via urine culture.

The urodynamic assessment was performed according to International Continence Society Standards [13], which involved water cystometry with 37°C normal saline solution at a filling rate of 50 mL/min. This is a medium filling used in clinical practice and in experimental studies, reserving a lower filling rate for neurogenic disorder. A 6F double-lumen Nelaton transurethral catheter was used for infusion and recording intravesical pressure, and a 16-channel intrarectal balloon catheter was used to record abdominal pressure.

Cystometry detrusor overactivity (DO) and bladder compliance (BC) defined as normal (>20 mL/cmH_2_O), impaired (10–20 mL/cmH_2_O), or poor (<10 mL/cmH_2_O) were recorded.

*Q*_max⁡_, detrusor pressure at opening (*P*_detOpen_), detrusor pressure at maximum flow (*P*_det⁡_ *Q*_max⁡_), and PVR were recorded.

BOO and detrusor contractility were assessed on pressure flow studies using Schafer's nomogram. Grades 0-1 bladder outlet conditions were considered unobstructed; Grade 2 is an equivocal score, and Grades 3–6 were considered obstructed. The nomogram was also used to classify detrusor strength as normal, weak, or very weak. Voiding by straining or detrusor contraction was also recorded.

At the end of the study, we measured the Valsalva leak point pressure (VLPP). Specifically, after filling the bladder to 150–200 mL, the catheter was removed and the Valsalva manoeuvers were repeated to measure abdominal pressure. The VLPP is defined as the lowest abdominal pressure that induces visible stress incontinence, and it assesses intrinsic sphincter deficiency (ISD).

### 2.4. Data Analyses

Data analyses were performed using tests for repeated nonparametric data (i.e., the Friedman and Cochran *Q* tests). The Bonferroni correction was applied to the Wilcoxon and McNamara tests for multiple post hoc comparisons. The *χ* ^2^ test was applied for trend data.

Correlations among variables were tested using Spearman's rho correlation coefficient.

Multivariate logistic regression models that incorporated the baseline parameters were fit to predict outcomes. The goodness of fit of these models (i.e., internal calibration) was checked using the Hosmer-Lemeshow test. Odds ratios (ORs) with 95% confidence intervals were also calculated.

Data are reported as the mean ± standard deviation. The level of significance was set at *P* < 0.05. All data analyses were performed using SPSS version 10.1.1 for Windows (SPSS, Chicago, IL, USA).

## 3. Results

### 3.1. Demographics

The mean age of the patients was 73.6 ± 3.1 yrs (median = 74.0 yrs, range = 67–79 yrs); the mean PSA value was 6.3 ± 3.0 ng/mL (median = 6.4 ng/mL, range = 2.4–14.3 ng/mL).

The mean prostate volume at baseline was 40.2 ± 15.6 mL (median = 38.5 mL, range = 16.0–52.0 mL).

Eleven participants (36.6%) were classified as clinical stage T1c, and the remaining participants were categorized as T2. A total of 21 participants had Gleason scores of ≤6 (70%), whereas 9 patients had scores of ≥7.

The mean treatment length was 113.7′ ± 38.8′ (median = 106.5′, range = 49′–240′); the mean time to spontaneous voiding was 13.2 ± 1.5 days (median = 12.8 days, range = 12–16 days).

The mean PSA nadir was 0.23 ± 0.55 ng/mL (median = 0.03 ng/mL, range = 0.00–2.66 ng/mL), which was reached in a median of 2.1 months (range = 1–3 months). At 6 months, the mean PSA was 0.47 ± 0.86 ng/mL (median = 0.18 ng/mL, range = 0.00–4.50 ng/mL). The 6-month positive prostate biopsy rate was 16.6% after one treatment.

### 3.2. Urinary Symptoms and Baseline Questionnaires

Table 1 presents the survey and continence status data.

The mean IPSS was 9.2 ± 5.9 (median = 9.5, range = 0–20). Seventeen patients (56.6%) showed moderate-to-severe lower urinary tract symptoms.

Twelve patients (40%) complained of preoperative abnormal sexual function, with a mean IIEF-5 score of 6 ± 7.7. However, only 58.4% of these patients showed an IIEF-5 score ≤16.

Six patients complained of urgency and urge incontinence, but none used pads or collecting devices.

### 3.3. Urodynamic Measures at Baseline

The mean *Q*_max⁡_ was 13.8 ± 5.3 mL/s (median = 13.0 mL/s, range = 5–23 mL/s), and the mean PVR was 31.8 ± 65.3 mL (median = 0 mL, range = 0–210 mL).

**Figure 1:**
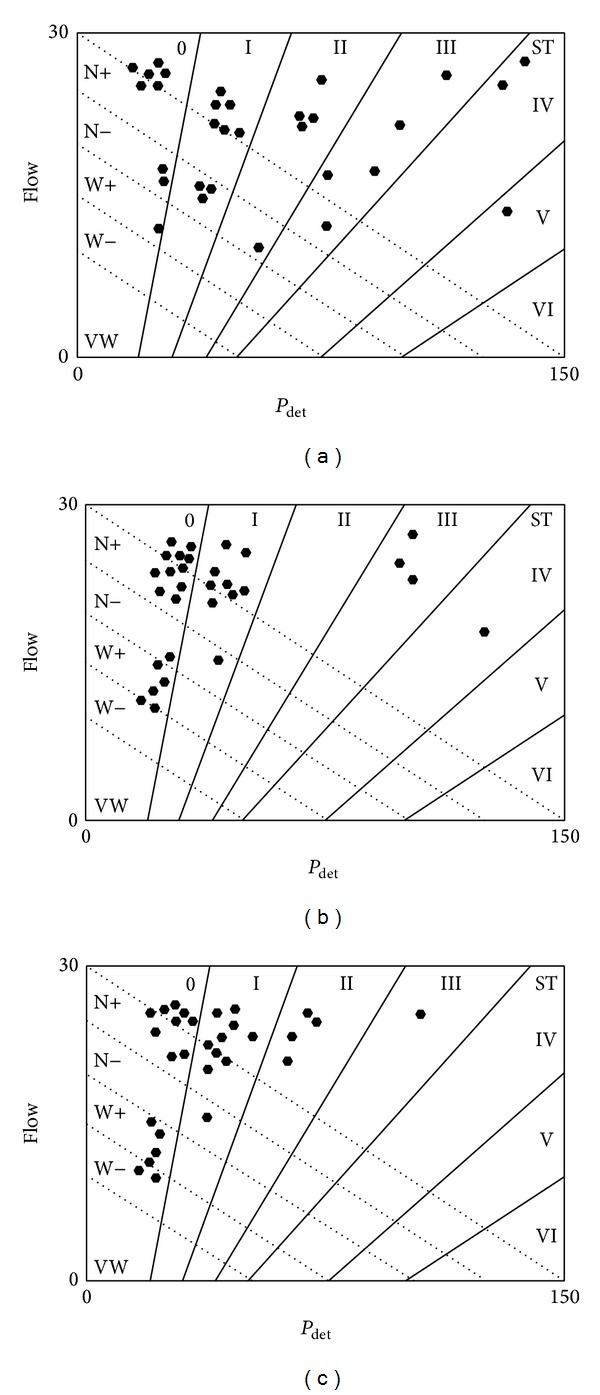
(a) Detrusor pressure at maximum detrusor pressure (*P*_detmax_) and maximum flow rate (*Q*_max⁡_), as assessed by the Schafer nomogram before HIFU. (b) Detrusor pressure at maximum detrusor pressure (*P*_detmax_) and maximum flow rate (*Q*_max⁡_), as assessed by the Schafer nomogram 3 months after HIFU. (c) Detrusor pressure at maximum detrusor pressure (*P*_detmax_) and maximum flow rate (*Q*_max⁡_), as assessed by the Schafer nomogram 6 months after HIFU.

Table 2 and Figure 1(a) show the urodynamic and Schafer's nomogram results, respectively.

DO was detected in 16 patients (53.4%); impaired or poor BC was detected in 9 patients (30%); and impaired detrusor contractility was detected in 13 patients (43.4%). BOO was found in 7 patients (23.4%).

The mean *P*_detOpen_ was 41.0 ± 28.9 cmH_2_O (median = 28.5 cmH_2_O, range = 16–136 cmH_2_O), and the mean *P*_det⁡_ *Q*_max⁡_ was 40.7 ± 24.3 cmH_2_O (median = 32.5 cmH_2_O, range = 6–110 cmH_2_O), which corresponds to a mean *Q*_max⁡_ of 13.9 ± 4.4 mL/s (median = 13.0 mL/s, range = 5–22 mL/s) and a mean PVR of 33.7 ± 71.2 mL (median = 30.4 mL, range = 0–190 mL).

Schafer's nomogram in Figure 1(a) shows impaired detrusor contractility with BOO in 2 patients (6.6%) and BOO plus strong detrusor contractility in 3 patients (10%).

The VLPP was positive in only one patient at an abdominal pressure of 65 cmH_2_O.

### 3.4. Three- and Six-Month Follow-Up Evaluations

#### 3.4.1. Urinary Symptoms and Questionnaires

Table 1 shows the questionnaires and continence status follow-up data.

The mean IPSS was 9.2 ± 5.9 and 8.5 ± 4.5 at 3 and 6 months, respectively, and no difference emerged compared with baseline (*P* = 0.576). Likewise, no differences emerged at 3 or 6 months among patients with mild-to-moderate LUT symptoms.

Of the patients who reported preoperative normal sexual function, 10 (83.3%) continued to report that they had no problems at the follow-up assessment. However, according to their IIEF-5 scores, 70% of these patients showed impaired sexual function. The differences at follow-up were not significant (*P* = 0.432).

No patients showed a de novo stress incontinence status, whereas 26.7% and 16.7% of patients suffered from urge incontinence at 3 and 6 months, respectively (*P* = 0.341). A significant increase in de novo urge incontinence was observed at 3 months compared with baseline (*P* = 0.04).

### 3.5. Urodynamic Study

The free uroflowmetry showed mean *Q*_max⁡_ scores of 15.0 ± 6.7 mL/s (median = 14.0 mL/s, range = 11–19 mL/s) and 16.9 ± 6.8 mL/s (median = 15.8 mL/s, range = 11–23 mL/s) at 3 and 6 months, respectively (*P* = 0.04); the mean PVR was 30.5 ± 54.3 mL (median = 0 mL, range = 0–180 mL) and 21.1 ± 33.4 mL (median = 10.0 mL, range = 0–40 mL) at 3 and 6 months, respectively (*P* = 0.06).

Three patients required urethral dilation at an early postoperative follow-up.

Table 2 and Figures 1(b)–1(c) show the urodynamic and Schafer's nomogram results at follow-up, respectively.

De novo DO was detected in 3 patients (10%) at the 3-month follow-up evaluation. A slight deterioration of BC was observed at 3 months. At the 6-month follow-up assessment, only 26.4% of patients showed an impaired BC. The mean maximum bladder capacity remained unchanged.

Detrusor contractility showed progressive (although not significant) improvement over time.

BOO was found in 3 patients (10%) at 3 months and 1 patient (3.4%) at 6 months (*P* = 0.04). These scores corresponded to mean *P*_detOpen_ scores of 30.3 cmH_2_O and 30.2 cmH_2_O and mean *P*_det⁡_ *Q*_max⁡_ scores of 33.1 cmH_2_O and 38.8 cmH_2_O at 3 and 6 months, respectively (*P*s = 0.01).

Mean *Q*_max⁡_ progressively increased from 3 to 6 months (*P* = 0.02).

Schafer's nomograms (Figures 1(b)–1(c)) associated with the 3- and 6-month follow-up assessments revealed that no patients had impaired detrusor contractility associated with BOO or BOO associated with strong detrusor contractility.

The VLPP was positive for two patients (abdominal pressures of 37 and 65 cmH_2_O) at 3 months and one patient (abdominal pressure = 73 cmH_2_O) at 6 months.

The 6-month IPSS score was positively correlated with *P*_detOpen_ (rho = 0.361, *P* = 0.05) but negatively correlated with *Q*_max⁡_ (rho = −0.657, *P* < 0.01).

The multivariate analysis revealed that only prostate volume (*P* = 0.02) and procedural length (*P* = 0.04) predicted the 6-month IPSS score.

The 6-month IIEF-5 score was positively correlated with the baseline IIEF-5 score (rho = 0.569, *P* < 0.01) but negatively correlated with age (rho = −0.457, *P* = 0.01); however, no correlations were found with regard to prostate volume and procedural length. A multivariate analysis revealed that only the baseline IIEF-5 score significantly predicted the 6-month IIEF-5 score (*P* = 0.01).

Six-month continence status was positively correlated with a strong voiding desire (rho = 0.382, *P* = 0.03) and urgency (rho = 0.373, *P* = 0.04) at urodynamic evaluation. A multivariate analysis revealed that prostate volume (*P* = 0.04) and procedural length (*P* = 0.02) predicted urge incontinence.

*P*_detOpen_ was positively correlated with *P*_det⁡_ *Q*_max⁡_ (rho = 0.789, *P* < 0.01) and obstructed voiding (rho = 0.384, *P* = 0.03) but negatively correlated with *Q*_max⁡_ (rho = −0.535, *P* < 0.01); however, no correlations were found with regard to age, prostate volume, or procedural length. *P*_det⁡_ *Q*_max⁡_ was negatively correlated with *Q*_max⁡_ (rho = −0.455, *P* = 0.01) but not with other variables.

## 4. Discussion

To our knowledge, present study is the first reporting the results of a prospective investigation on urinary symptoms and urodynamic findings among patients with prostate cancer treated using HIFU. Our results demonstrate that this procedure is safe with regard to LUT function.

HIFU has been investigated since 1987 with regard to the prostate gland. In 1993, Madersbacher et al. [14] presented the results of a Phase II study on the safety and efficacy of tissue ablation using HIFU among 36 patients with symptomatically benign prostatic hyperplasia (BPH). These authors demonstrated that HIFU reduced urinary symptoms and increased urinary flow. Later, the same group studied the 3- to 6-month urodynamic changes induced by HIFU among patients with BPH. The use of HIFU for BPH relieving was an interesting technique but characterized by the treatment of various amounts of prostatic tissue surrounding the urethra. In case of prostate cancer and in whole-gland therapy, urodynamic impact upon lower urinary tract is variably reported. Moreover, the functional outcomes after HIFU are influenced by the use of different devices, different surgical approaches, and follow-ups.

Many centers perform a TURP immediately prior to HIFU, a few weeks before HIFU, or immediately after HIFU [15]. These approaches reduce the risk of prolonged urinary retention [16] and permit the treatment of high-volume prostates [17–19]. Although helpful, the addition of TURP partially reduces the mini-invasivity of the procedure; moreover, TURP requires an adjunctive anaesthesia.

Interestingly, other experiences emphasize the fact that the rates of long-term BOO and urethral stenosis do not decrease when TURP is conducted in conjunction with HIFU. For example, Ganzer et al. [20] showed that the incidence of postoperative BOO was 28.3%; furthermore, 16.9% of patients reported stress incontinence that required surgery for 0.7% of all patients. Other studies showed that the incidence of postoperative BOO was similar after comparing HIFU alone with HIFU + TURP before (21.9% versus 17.9%, resp.) and after HIFU + contemporary TURP (34.3%) [21].

The primary advantages of HIFU alone are reducing invasivity and costs; furthermore, HIFU alone might alleviate LUT symptoms. The major disadvantages of HIFU alone are the impossibility of treating high-volume prostate glands; moreover, a higher incidence of early postoperative LUT symptoms is observed.

Neoadjuvant antiandrogen therapy is a valid alternative to TURP to reduce prostate volume [12, 19]. Evaluating the efficacy of this approach is beyond the scope of current study, but it is a valid alternative when prostate volume limits the use of HIFU.

HIFU affects LUT in the early postoperative period. Our study confirmed that a high proportion of patients (26.7%) presented urgency and urge incontinence; however, these symptoms were also observed in 20% of patients before surgery, suggesting that they can be attributed to surgical damage to the bladder neck and prostatic urethra in a small percentage of patients. The presence of urine, edema, and debris in the proximal urethra activates afferent circuits from the prostate to the bladder, inducing involuntary detrusor contractions [22]. At 6-month follow-up assessment, when sloughing and edema were resolved, these symptoms reduced in most patients. The urodynamic study of storage phase showed a correspondence with increased DO and impaired BC at 3 months showing improvement at 6-month follow-up.

Not all HIFU candidates presented preoperative LUT symptoms suggestive of BOO. According to the IPSS, only 56.6% of our patients had mild-to-moderate symptoms. The *Q*_max⁡_ at free uroflowmetry was >15 mL/s in 40% of patients. The pressure/flow study showed that 76.6% of patients had normal bladder outlet.

Based on the pressure/flow study and Schafer's nomogram (Figures 1(b)–1(c)), most patients with preoperative BOO will have unobstructed flows. The *P*_detOpen_ and *P*_det⁡_ *Q*_max⁡_ significantly decreased at follow-up, corresponding to increased *Q*_max⁡_ and reduced PVR. However, despite the improvements in pressure and flow, the 6-month IPSS revealed that a discrete proportion of patients continued to suffer from mild-to-moderate symptoms, mostly related to irritation. Based on the multivariate analysis showing that prostate volume and procedure length predicted the 6-month IPSS, we hypothesize that patients with high-volume prostates require more time to relieve edema and debris passage and resolution of irritative and obstructive symptoms. Note that three patients with high IPSSs required urethral dilation. This finding matches that of other studies demonstrating that high power and the overlap of treated areas (e.g., for high-volume prostates) produce higher rates of postoperative complications [23].

All types/degrees of urinary incontinence have been reported in 1%–34.3% of patients following HIFU [5]. It is unclear whether an association exists among stress incontinence, HIFU, and TURP; however, previous experience has shown that the rate of urinary incontinence is significantly lower among patients receiving TURP + HIFU compared with those receiving HIFU alone [24]. In our experience (present and previous one [11]), no patients experienced isolated stress urinary incontinence except those who received a second HIFU session. The VLPP was low in only one patient at 3-month follow-up. De novo urge incontinence developed in 6.7% of patients at 3 months.

The present study has several limitations. It is a single-arm study without any comparison with a group of patients treated with HIFU + TURP; furthermore, only a small number of patients were included, and the follow-up was short.

## 5. Conclusions

Lower urinary tract function following HIFU for prostate cancer should be adequately assessed by symptoms' analysis and urodynamic studies.

In the long term, preoperatively impaired bladder outlets, early postoperative detrusor overactivity, decreased bladder compliance, and patient-reported urgency and urge incontinence leave the place to a progressive increasing in bladder compliance, improved bladder outlet, a significantly increasing maximum flow rate, and reduced PVR.

The current urodynamic study confirmed previous data, thereby demonstrating that HIFU alone can be delivered safely.

## Figures and Tables

**Table 1:** Subjective data relative to International Prostate Symptoms Score IPSS, International Index Erectile Function 5 IIEF, continence status, and urgency.

	Baseline			3 months			6 months			*P*
	Number of pts.	%	Mean	Number of pts.	%	Mean	Number of pts.	%	Mean	
IPSS										
	30	100	9.2	30	100	9.2	30	100	8.5	0.576
Normal	13	43.4		13	43.4		17	56.6		
Mild-moderate	17	56.6		17	56.6		13	43.4		
IIEF										
Potent	12			10			10			0.432
Normal	5	41.6		3	30.0		3	30		
Poor	7	58.4		7	70.0		7	70		
Continence										
Complete	24	80		22	73.3		25	83.3		0.341
Urge	6	20		8	26.7*		5	16.7		
Stress	0	0		0	0		0	0		
Urgency	20	66.6		24	80*		17	56.6*		0.04*

*P < 0.05.

**Table 2:** Data relative to urodynamic studies. Main patterns of storage and voiding phases.

	Baseline			3 months			6 months			*P*
	Number of pts.	%	Mean	Number of pts.	%	Mean	Number of pts.	%	Mean	
Detrusor										
Normal	14	46.6		11	36.7		14	46.6		0.184
Overactive	16	53.4		19	63.3		16	53.4		
Bladder compliance (mL/cmH_2_O)										
Normal	21	70.0		18	60.0		22	73.3		
Impaired	8	26.6		12	40.0		7	23.3		0.876
Poor	1	3.4		0	0		1	3.4		
Voiding desire (mL)										
First	30		99.6	30		94.2	30		98.1	0.837
Strong	30		255.2	30		255.1	30		250.9	0.955
Max. capacity (mL)	30		328.1	30		311.1	30		323.2	0.389
Bladder outlet										
Normal	23	76.6		27	90		29	96.6*		0.04*
Obstructed	7	23.4		3	10		1	3.4*		
*P*_detOpen⁡_ (cmH_2_O)	30		41.0	30		30.3*	30		30.2*	0.01*
*P*_det⁡_ *Q*_max⁡_ (cmH_2_O)	30		40.7	30		33.1*	30		38.8*	0.01*
Detrusor contractility										
Normal	17	56.6		23	76.7		23	76.7		0.174
Weak	10	33.4		7	23.3		7	23.3		
Strong	3	10		0	0		0	0		
Voiding										
Without straining	15	50		17	56.6		16	53.4		0.189
With straining	15	50		13	43.4		14	46.6		
Volume at uroflowmetry	30		292.5	30		169.0*	30		247.0	0.03*
Qmax (mL/s)	30		13.9			16.4*			16.9*	0.02*
PVR (mL)	30		31.8			14.0			10.0*	0.05*
VLPP (cmH_2_O)	0			1		37	0			

*P < 0.05.
